# Regulatory roles and molecular mechanisms of N6-methyladenosine (m6A) modification in ischemia-reperfusion injury

**DOI:** 10.7717/peerj.21875

**Published:** 2026-11-06

**Authors:** Di Zhang, Wenjuan An, Xing Zhang, Yuhang Han, Guoxin Zhang, Lingyun Zhang

**Affiliations:** 1 The First Clinical Medical College of Gansu University of Chinese Medicine, Lanzhou, Gansu, China; 2 School of Basic Medical Sciences, Gansu University of Chinese Medicine, Lanzhou, Gansu, China; 3 Department of Anesthesiology, Gansu Provincial Hospital of Traditional Chinese Medicine, Lanzhou, Gansu, China

**Keywords:** m6A modification, Ischemia-Reperfusion, RNA methylation, Molecular mechanism of multi-organ injury

## Abstract

**Background:**

N6-methyladenosine (m6A) represents a prevalent internal epitranscriptomic modification of mRNA that influences post-transcriptional gene regulation. Clinically, ischemia-reperfusion (I/R) injury contributes to tissue damage in acute kidney injury, ischemic stroke, and myocardial infarction, particularly after blood flow is restored.

**Methods:**

We searched Google Scholar, Web of Science, and PubMed for literature published through May 2026. The retrieval focused on articles detailing how m6A effector proteins—specifically writers, erasers, and readers—mediate I/R pathogenesis within hepatic, renal, cardiac, and cerebral tissues.

**Results:**

m6A-associated regulation influences RNA metabolism during I/R injury. Context-dependent changes in m6A regulators and target transcripts are linked to oxidative stress, inflammatory cascades, and specific programmed cell death pathways, including ferroptosis and pyroptosis, across various organ systems.

**Conclusion:**

Profiling these organ-specific m6A molecular networks may inform future research on targeted epitranscriptomic interventions for IRI.

## Introduction

Ischemia-Reperfusion Injury (IRI) is widely observed in clinically vital organs such as the heart, liver, and kidney during surgical procedures, transplantation, and acute disease processes (Sánchez-Hernández et al., 2020). While rapid reperfusion remains the primary clinical intervention to salvage ischemic tissue, the subsequent restoration of oxygenated blood paradoxically initiates detrimental molecular cascades. This secondary insult drives mitochondrial failure, severe oxidative stress, and inflammatory cascades, ultimately culminating in programmed cell death and compromised patient outcomes (Abela & Homer-Vanniasinkham, 2003). In the context of epitranscriptomics, N6-methyladenosine (m6A) RNA methylation has emerged as a primary post-transcriptional regulator. Understanding these dynamic RNA modifications provides an alternative mechanistic framework for evaluating ischemia-reperfusion (I/R)-induced cellular damage and elucidating the underlying molecular pathways. As the dominant epigenetic modification in eukaryotic messenger RNA, the m6A regulatory system relies on a dynamic interplay among specific methyltransferases (METTL3, METTL14, and WTAP), demethylases (FTO and ALKBH5), and reader proteins (including the YTH family). Together, these effector proteins orchestrate critical aspects of RNA metabolism, encompassing mRNA splicing, nuclear transport, stability, and translation (Wang et al., 2022a). Emerging evidence indicates substantial fluctuations in m6A profiles across various *in vivo* IRI models. By modulating key signaling cascades, these epitranscriptomic shifts directly influence oxidative stress, autophagic flux, and specific programmed cell death execution (*e.g*., ferroptosis, pyroptosis, and cuproptosis) (Yao et al., 2020). Decoding these dynamic methylation patterns and their downstream effectors may help identify candidate biomarkers and therapeutic targets for future validation. Ultimately, elucidating these RNA epigenetic pathways may guide future investigation of targeted interventions against ischemia-reperfusion damage. Given the rapid accumulation of conflicting data across different organs, this timely review is critically needed to consolidate these fragmented findings into a cohesive framework. The intended audience includes basic medical researchers, clinical physicians, and medical students focusing on molecular pathology, helping them understand the relevance of m6A dynamics to cross-disciplinary areas such as targeted critical care therapies and organ transplantation. This article is a narrative synthesis informed by a structured literature search rather than a formal systematic or scoping review. It provides a cross-organ, target-resolved comparison of m6A regulator-RNA-reader relationships and distinguishes mechanistic I/R evidence from profiling, bioinformatic, and non-IRI observations.

## Methods

This review was conducted to provide a structured narrative synthesis of the regulatory functions and molecular mechanisms of m6A modification in ischemia-reperfusion injury (IRI) across multiple organs. The methodology encompassed literature search, study selection, data extraction, and synthesis of evidence.

### Literature search strategy

We retrieved relevant publications indexed through May 2026 from Google Scholar, PubMed, and Web of Science without initial date restrictions. For PubMed, free-text queries were combined with Medical Subject Headings (MeSH); the corresponding free-text concepts were adapted for Web of Science and Google Scholar. Primary search terms encompassed “N6-methyladenosine” or “m6A” in combination with “ischemia-reperfusion injury.” Specific organ-related queries included “myocardial infarction,” “stroke,” “liver transplantation,” and “acute kidney injury,” alongside specific cell death modalities such as “pyroptosis,” “ferroptosis,” and “autophagy,” as well as key effector proteins like “METTL3,” “FTO,” and “YTHDF.” To broaden coverage, no initial publication date or geographical restrictions were applied, and the reference lists of retrieved articles were manually screened to avoid inadvertent omission of relevant studies. The core Boolean search string was: (“N6-methyladenosine” OR “m6A”) AND (“ischemia-reperfusion injury” OR “ischemia reperfusion”) AND (“heart” OR “myocardial” OR “brain” OR “cerebral” OR “liver” OR “hepatic” OR “kidney” OR “renal”), combined with the effector- and cell-death-specific terms listed above. Per-database record counts were not prospectively logged.

### Literature screening and eligibility criteria

We initially evaluated records by analyzing titles and abstracts, followed by a comprehensive full-text assessment. Inclusion required studies to meet the following criteria: (1) presentation of original *in vitro*, *in vivo*, or clinical data; (2) investigation of m6A effector proteins—writers, erasers, or readers—during the pathogenesis of cerebral, cardiac, hepatic, or renal IRI; (3) identification of specific signaling networks or molecular targets; and (4) publication in either English or Chinese. Studies not focused on IRI, non-research articles, and studies in which m6A was not a primary focus were excluded from the organ-specific mechanistic synthesis; reviews and non-IRI studies were retained only for background or explicitly labeled hypothesis-generating context.

### Data extraction and synthesis

For each eligible study, the extracted information included: author(s), publication year, organ/system studied, experimental model (*e.g*., animal model, cell line), key m6A regulatory proteins investigated (*e.g*., METTL3, FTO, YTHDF2), identified target genes or non-coding RNAs, affected signaling pathways (*e.g*., Akt/mTOR, NF-κB, HIF-1α), associated cellular processes (*e.g*., cell death modalities, inflammation, mitochondrial dynamics), and main findings regarding the functional impact (protective or detrimental) of m6A modulation. Data synthesis was performed thematically. Evidence was organized by target organ (heart, brain, liver, kidney) and further categorized according to the type of m6A regulatory protein involved and the downstream mechanistic pathways. Cross-organ comparisons were made to identify common patterns and organ-specific particularities in m6A regulation during IRI.

## Results

### Basic overview of m6A

#### Concept and characteristics of m6A

While transcriptomes harbor over a hundred distinct chemical modifications across various RNA species (Jonkhout et al., 2017), methylation at the N6 position of adenosine (m6A) dominates the mammalian epitranscriptomic landscape. Constituting roughly half of all methylated ribonucleotides, m6A typically occurs at three to five sites per mRNA transcript. This modification preferentially targets the RRACH consensus motif (R = A/G, H = A/C/U), exhibiting marked enrichment near stop codons, within 3′-untranslated regions (3′-UTRs), and across long internal exons (Yang et al., 2020). Historically viewed as a static structural feature, the identification of specific methyltransferase complexes (*e.g*., METTL3/METTL14/WTAP) and demethylases (FTO and ALKBH5) fundamentally shifted this paradigm (Fang et al., 2021). These discoveries established m6A as a reversible, dynamically regulated epigenetic mechanism with profound implications for determining cell fate and physiological responses.

#### The proteomic components of m6A

The dynamic m6A landscape relies on a tripartite enzymatic system: writers (methyltransferases), erasers (demethylases), and readers (recognition proteins). Functioning collaboratively, these effectors govern the entire RNA lifecycle. Specifically, writers deposit the methylation mark, erasers catalyze its removal, and readers decode the modification to execute downstream functions. This coordinated regulation dictates alternative splicing site selection, nuclear-to-cytoplasmic export efficiency, ribosomal binding affinity during translation, and overall transcript decay rates, thereby maintaining strict cellular RNA homeostasis (Fig. 1).

**Figure 1:**
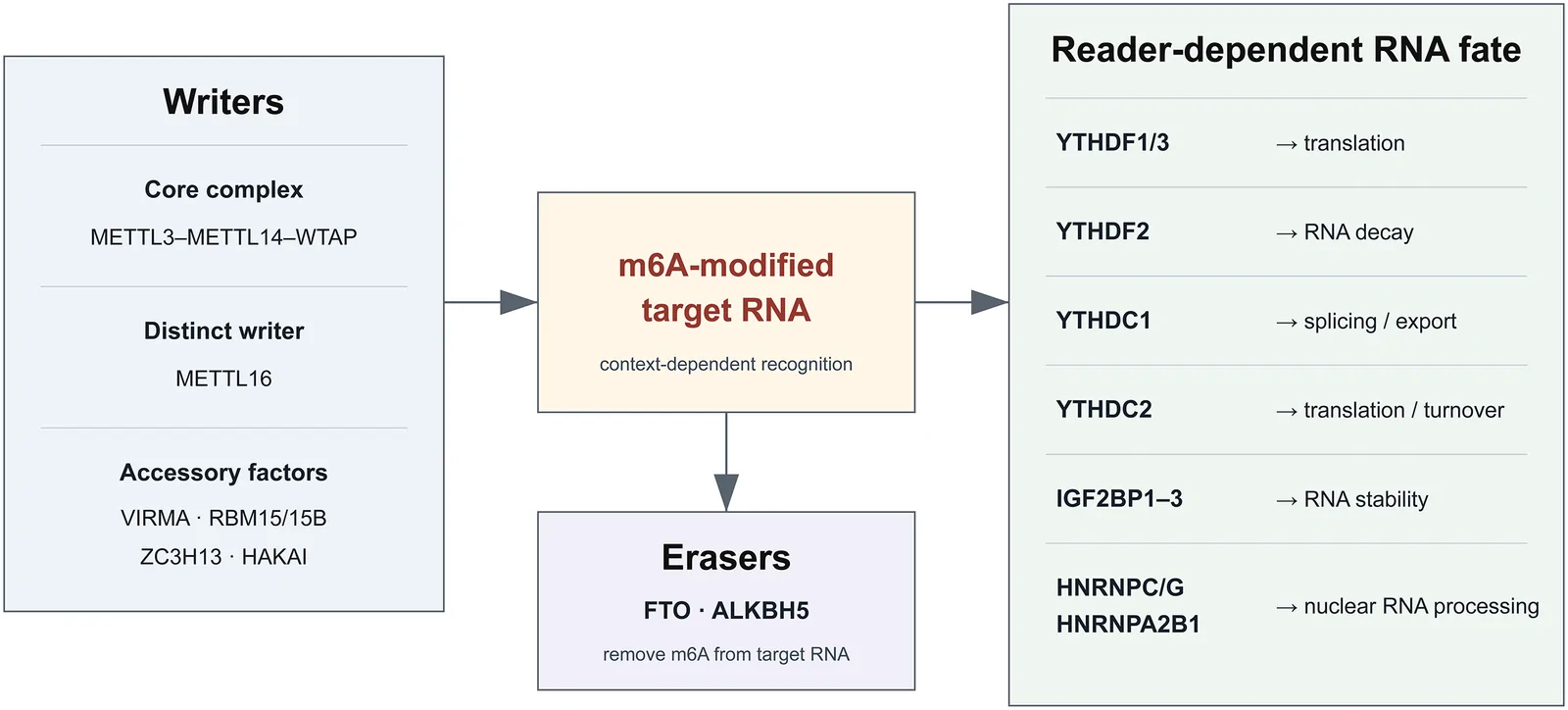
Simplified organization of the m6A writer–eraser–reader machinery. The writer module includes the METTL3–METTL14–WTAP core complex, the distinct writer METTL16, and the accessory factors VIRMA/KIAA1429, RBM15/15B, ZC3H13, and HAKAI. Writers deposit m6A on target RNAs, whereas FTO and ALKBH5 remove the modification. Reader proteins direct context-dependent RNA fates: YTHDF1/3 are associated with translation, YTHDF2 with RNA decay, YTHDC1 with splicing and export, YTHDC2 with translation and turnover, IGF2BP1–3 with RNA stability, and HNRNPC/G and HNRNPA2B1 with nuclear RNA processing.

##### m6A “writers”

m6A writers, also referred to as encoders, constitute a multi-component methyltransferase complex that catalyzes the m6A modification process. This methyltransferase complex primarily comprises various components, such as METTL14, METTL3, Wilms’ tumor 1-associating protein (WTAP), METTL16, the human homolog of the *Drosophila* Virilizer protein (KIAA1429/VIRMA), RNA-binding motif protein (RBM15/15B), zinc finger CCCH domain-containing protein (ZC3H13), zinc finger protein (ZFP), and the Cbl proto-oncogene E3 ubiquitin ligase (HAKAI) (Schöller et al., 2018). Among these, METTL3 is an S-adenosyl-L-methionine (SAM)-binding protein and serves as a crucial component of the m6A methyltransferase complex (MTC), exhibiting high conservation in eukaryotes such as yeast and humans. METTL14 is another active component of the m6A MTC. METTL3 and METTL14 co-localize within nuclear speckles, forming a stable heterodimer complex at a 1:1 ratio, and they synergistically induce m6A formation. They constitute a stable heterodimeric nuclear complex wherein METTL3 primarily functions as the catalytic core, while METTL14 provides structural support for METTL3 (Wang et al., 2020). WTAP is an adaptor protein that interacts with both METTL3 and METTL14 and plays a key role in their localization to nuclear speckles. METTL16 is a newly discovered, independent RNA methyltransferase (Jiang et al., 2021); it is a conserved U6 small nuclear RNA (snRNA) methyltransferase capable of regulating cellular S-adenosylmethionine (SAM) levels. RBM15/15B interacts with METTL3 in a WTAP-dependent manner and assists in recruiting the complex to specific sites for methylation. Furthermore, several other proteins, such as ZC3H13, Vir-like m6A methyltransferase-associated protein (VIRMA/KIAA1429), and others, are also involved in the m6A methylation process and are present within the m6A methyltransferase complex (Wang et al., 2022a).

##### m6A “erasers”

Reversibility of the methylation mark is executed by the ALKB family demethylases, namely FTO and ALKBH5 (Li et al., 2021). FTO operates *via* an Fe(II)- and α-ketoglutarate (α-KG)-dependent oxidative demethylation pathway, preferentially targeting intronic pre-mRNA regions adjacent to alternative splice sites and polyadenylation signals. Conversely, ALKBH5 directly strips the methyl group without an intermediate oxidative state, similarly utilizing Fe^2+^ and α-KG as cofactors within nuclear speckles (Choy et al., 2022).

##### m6A “readers”

The functional output of m6A methylation is ultimately decoded by specialized RNA-binding proteins. The YT521-B homology (YTH) domain family constitutes the primary reader class. Specifically, YTHDF1 and YTHDF3 synergize with eIF3 to augment translational efficiency (Wu et al., 2020), whereas YTHDF2 selectively recruits the CCR4-NOT deadenylase complex to accelerate mRNA decay. Within the YTH family, YTHDC1 participates in RNA splicing and nuclear export, whereas YTHDC2 regulates translation and RNA turnover. Beyond the YTH family, other proteins like IGF2BPs and diverse HNRNPs (C/G/A2B1) dynamically influence transcript stability, splicing, and translation based on specific cellular contexts (Xu et al., 2022).

### m6A and ischemia-reperfusion injury

#### Regulatory mechanisms of m6A in myocardial ischemia-reperfusion injury

The diverse regulatory roles of m6A modification in ischemia-reperfusion injury across multiple organs, including the heart, brain, liver, and kidney, are summarized in Fig. 2. In the field of myocardial ischemia-reperfusion injury (MIRI) research, m6A modification has emerged as a critical focus. Current studies primarily concentrate on identifying key regulatory factors and their downstream signaling pathways. Using GEO expression profiles from patients with acute myocardial infarction, the study identified seven candidate m6A regulators—IGF2BP1, FTO, RBM15, METTL3, YTHDC2, FMR1, and HNRNPA2B1—and used them to construct a diagnostic nomogram. Consensus clustering defined two m6A-related AMI subtypes. High METTL3 expression was associated with higher activated CD4+ T-cell and type 2 helper T-cell abundance and lower CD56bright natural killer-cell, macrophage, monocyte, natural killer-cell, and type 17 helper T-cell abundance. These bioinformatic findings identify candidate markers for AMI but do not establish a causal mechanism in myocardial I/R injury (Wang et al., 2022c).

**Figure 2:**
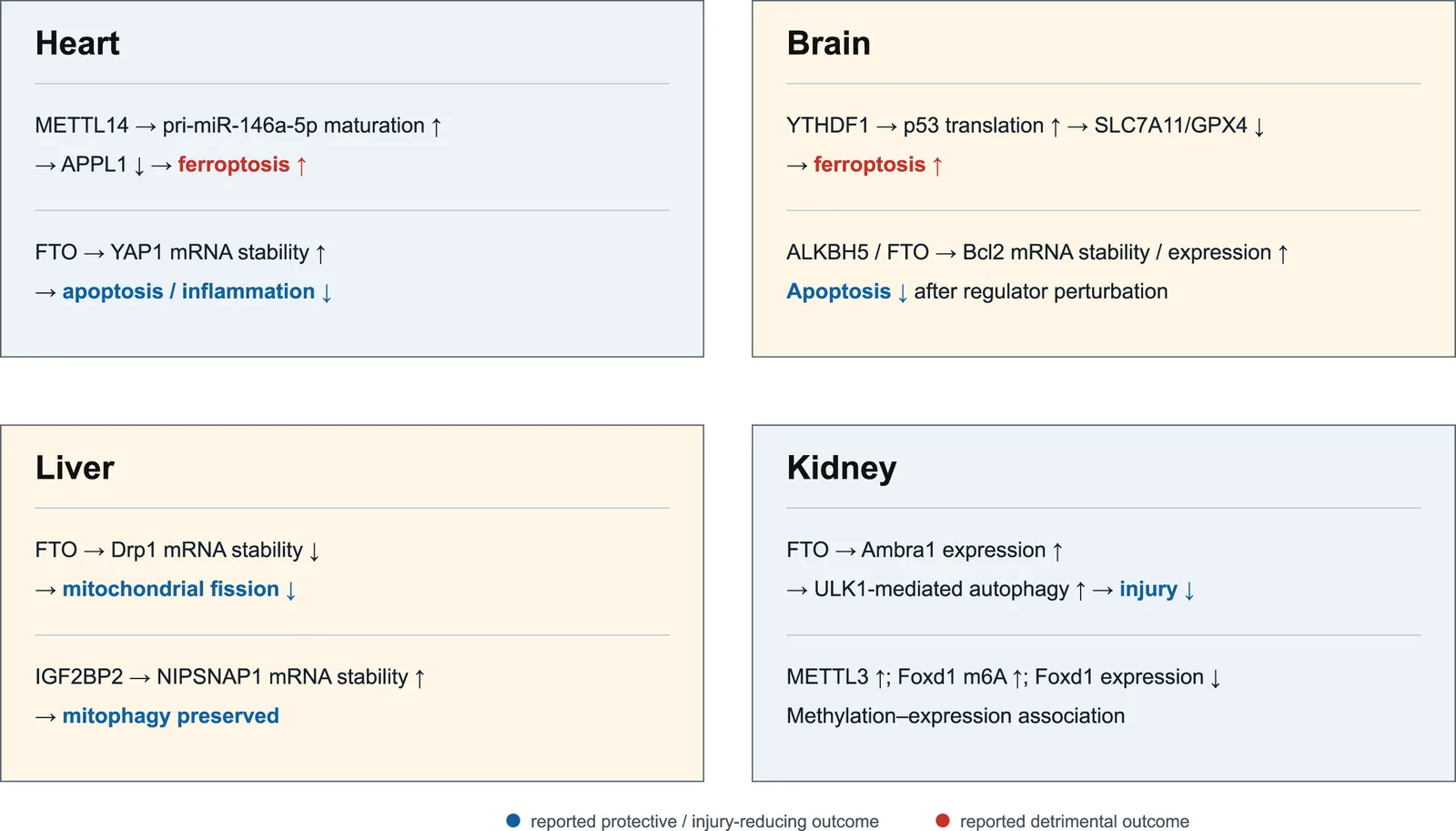
Representative organ-specific m6A regulatory axes in ischemia–reperfusion injury. The four panels show cardiac, cerebral, hepatic, and renal examples. Each panel presents two representative regulator–RNA–outcome relationships. Blue and red outcome text indicate reported protective and detrimental effects, respectively. Rightward arrows show the sequence of the reported relationships, whereas upward and downward arrows indicate increases and decreases, respectively.

Regarding lncRNAs, *Snhg1* expression is downregulated under hypoxia/reoxygenation (H/R) conditions. Its overexpression can significantly inhibit cell apoptosis, reduce mitochondrial ROS production, and improve membrane potential. WTAP can increase the m6A modification on *Snhg1* (a direct m6A substrate), promoting its degradation *via* YTHDF2. This indicates that WTAP-mediated m6A modification regulates the MIRI process through the *Snhg1*/*miR-361-5p* (a downstream effector not reported to be directly m6A-modified)/OPA1 axis (Liu et al., 2024a). Furthermore, studies have found that METTL14 is significantly upregulated during I/R injury. Its knockdown significantly alleviates cardiomyocyte apoptosis and necrosis, reduces oxidative stress product ROS, increases the activity of the antioxidant enzyme SOD, and simultaneously inhibits the secretion of pro-inflammatory cytokines IL-6 and IL-1β (Wu et al., 2024).

In MIRI rats, overexpression of YTHDF2 significantly improves cardiac function, reduces infarct size, and inhibits mitophagy. However, experiments demonstrated that BNIP3 overexpression can abolish the protective effect of YTHDF2 (Cai et al., 2023). Another study showed that METTL3 expression is significantly elevated in myocardial I/R rats and in cells subjected to oxygen-glucose deprivation/reoxygenation (OGD/R). Silencing METTL3 markedly improves cardiac function, alleviates myocardial pathological damage, and significantly reduces NLRP3 expression, caspase-1 activity, GSDMD-N cleavage, and the release of IL-1β/IL-18, supporting a pro-pyroptotic role for METTL3 in this model (Wang et al., 2023b).

A literature review by Liu et al. (2024b) pointed out that m6A levels are significantly elevated in both MIRI and doxorubicin-induced cardiotoxicity (DIC). Among the regulatory enzymes, methyltransferases such as METTL3 and WTAP promote injury, while demethylases like FTO and ALKBH5 exert protective effects. m6A influences cardiomyocytes by regulating the mRNAs of target genes such as *TFEB*, *YAP1*, *FOXO1*, and *TLR4*, thereby affecting downstream signaling pathways (Liu et al., 2024b). Ke et al. (2022) reported that FTO was downregulated in myocardial I/R tissue and H/R-treated cardiomyocytes; FTO overexpression reduced apoptosis and inflammatory-factor release in H/R-treated cardiomyocytes, and FTO-mediated demethylation was reported to increase YAP1 mRNA stability and expression. Furthermore, knocking down WTAP significantly improves cell proliferation and suppresses apoptosis and inflammatory responses (Wang et al., 2023a).

Aging hearts exhibit unique characteristics in maintaining m6A homeostasis during I/R injury, which is attributed to age-dependent downregulation of FTO. This suggests that FTO may counteract the decline in m6A levels caused by reduced METTL3 (Su et al., 2021). Another study found that Igf2bp2 recognizes Mettl3-mediated m6A modification to enhance the stability of *Klf6* mRNA. Klf6 directly activates Acsl4 expression, and Acsl4 overexpression can reverse the protective effect of Klf6 deficiency. This establishes the damaging role of the “m6A-Klf6-Acsl4” signaling axis in myocardial I/R injury (Qiu, Yan & Liu, 2023).

Zhao & Li (2024) discovered that METTL14 enhances the m6A modification on *pri-miR-146a-5p* (a direct m6A substrate), thereby increasing mature *miR-146a-5p* levels. *miR-146a-5p* directly targets and suppresses APPL1 transcription, subsequently exacerbating H/R-induced cardiomyocyte ferroptosis. Cheng et al. (2022) innovatively designed a surface-modified ferritin nanocage named HSSS. Loaded with the ALKBH5 inhibitor IOX1, it efficiently targets the lesion area both *in vitro* and *in vivo*, significantly reducing myocardial enzyme levels, decreasing infarct size, and improving cardiac function. The role of ALKBH5 in cardiac IRI has also recently begun to be defined through direct mechanistic studies. A study found that Alkbh5 promotes Ythdf1 expression through demethylation, thereby facilitating Fth1 translation and suppressing ferroptosis in a rat myocardial I/R model (Yin & Liu, 2025). This study supports a protective role in the tested cardiac model; however, the benefit reported with targeted delivery of the ALKBH5 inhibitor IOX1 should be interpreted as an intervention result rather than evidence that endogenous ALKBH5 inhibition is uniformly protective (Cheng et al., 2022; Yin & Liu, 2025).

A review emphasizes that mitochondrial dysfunction is a key driver of I/R injury, encompassing four pillars: energy depletion, ROS burst, calcium overload, and mitochondrial permeability transition pore (mPTP) opening. This provides essential background supporting research into m6A regulation of mitochondrial quality control (Kuznetsov et al., 2019).

#### Regulation and mechanisms of m6A modification in cerebral ischemia-reperfusion injury

Current research on the role of m6A modification in regulating cerebral ischemia-reperfusion injury (CIRI) is rich and diverse, encompassing multiple dimensions such as non-coding RNA regulation, acupuncture intervention, and cell death mechanisms.

At the cellular level, Chokkalla et al. (2019) were among the first to discover a significant increase in m6A levels in the brain during ischemic stroke. Concurrently, the m6A demethylase FTO showed a marked decrease at the mRNA and protein levels as well as in enzymatic activity, with the enzyme primarily localized to neurons (Chokkalla et al., 2019). A subsequent rat CIRI profiling study similarly reported increased global m6A and decreased FTO expression, supporting an association between reduced FTO and post-ischemic m6A accumulation (Shao et al., 2022).

Several studies investigating acupuncture found that the “Xingnao Kaiqiao” (awakening the brain and opening the orifices) acupuncture method can specifically regulate FTO, reduce the m6A methylation modification of ACSL4 mRNA, thereby inhibiting its expression and ferroptosis, ultimately alleviating CIRI and protecting the brain (Liu et al., 2026). Another technique, eye acupuncture therapy, was shown to alleviate neurological dysfunction caused by CIRI with increased angiogenesis in the cerebral cortex and changes in the METTL3/m6A/HIF-1α/VEGF-A signaling axis (Pu, Wang & Chu, 2025). Both findings remain preliminary because each derives from a single rat study without independent replication.

From the perspective of molecular mechanism research, Xu et al. (2020a) found that m6A levels increased significantly after cerebral ischemia, accompanied by upregulated Alkbh5 expression and downregulated FTO expression, while overexpression of FTO had a protective effect. Alkbh5/Fto perturbation affected *Bcl2* mRNA stability and protein expression; direct demethylation of Bcl2 mRNA was not demonstrated (Xu et al., 2020a). Furthermore, METTL3 overexpression can effectively inhibit the expression of lncRNA *CRNDE* (a direct m6A substrate), thereby reducing cell apoptosis, lowering levels of pro-inflammatory factors TNF-α, IL-1β, and IL-6, increasing the secretion of the anti-inflammatory factor IL-10, and enhancing autophagic activity (Yu et al., 2024). METTL14 promotes cuproptosis in hippocampal neurons by stabilizing *FDX1* mRNA *via* m6A modification, exacerbating cerebral IRI (Ding et al., 2025).

YTHDF1 expression increases after I/R, and knocking down YTHDF1 significantly alleviates oxidative stress, reduces lipid peroxidation and Fe2+ accumulation, restores SLC7A11/GPX4 expression, and decreases infarct volume (Chang et al., 2025). METTL3 promotes the degradation of lncRNA *AU020206* (a direct m6A substrate) by increasing its m6A modification; lncRNA *AU020206* binds to YTHDC2 and promotes the degradation of *SLC7A11* mRNA, weakening its antioxidant capacity; while silencing *AU020206* or overexpressing METTL3 stabilizes SLC7A11/GPX4 expression, significantly reducing neuronal apoptosis and ferroptosis (Zhang et al., 2025).

METTL3 can also enhance the stability of *ABHD11-AS1* (a direct m6A substrate) in an m6A-dependent manner, relieving the inhibition of HIF1AN by *miR-1301-3p* (a downstream effector, not itself reported to be directly m6A-modified), thereby suppressing the neuroprotective HIF-1α/Nox4 pathway (Huang et al., 2024). Additionally, METTL3 overexpression can enhance the m6A modification of *pri-miR-335* (a direct m6A substrate), promoting its maturation, which upregulates stress granule (SG) formation and inhibits apoptosis. Mature *miR-335* targets and degrades *Erf1* mRNA, relieving its inhibition of translational stalling, thereby promoting SG assembly (Si et al., 2020).

Research by Liu et al. (2024d) revealed that catalpol regulates the m6A modification of Beclin-1 through the NRF1/KAT2A/METTL3 axis, thereby inhibiting excessive autophagy and alleviating neuronal damage. m6A modification promotes blood-brain barrier (BBB) disruption in cerebral I/R injury by enhancing MMP3 expression, establishing a functional link for the m6A-MMP3 axis in this process (Liang et al., 2023).

These related studies have not only constructed a transcriptome-wide atlas of m6A in CIRI but have also systematically revealed that m6A modification, by regulating various molecular types such as lncRNAs, miRNAs, transcription factors, and structural proteins, is extensively involved in a series of key pathological processes including neuroinflammation, autophagy, ferroptosis, cuproptosis, and blood-brain barrier integrity.

### Regulation and mechanisms of m6A modification in hepatic ischemia-reperfusion injury

Extensive research has delved into the complex regulatory roles of m6A in hepatic ischemia-reperfusion injury (HIRI) from multiple perspectives, including mitochondrial dynamics, immune cell infiltration, autophagy, and transcriptome-wide profiling.

During ischemia-reperfusion (I/R), the continuous accumulation of reactive oxygen species (ROS) leads to a significant decrease in DHX58 expression. Deficiency of DHX58 exacerbates liver injury and activates the ferroptosis pathway. Specifically, DHX58 recruits YTHDC2 *via* its CTD domain. YTHDC2 recognizes the m6A modification on glutathione peroxidase 4 (*Gpx4*) messenger RNA (mRNA), thereby enhancing the translation efficiency of Gpx4 and ultimately increasing GPX4 protein expression. Interferon can reverse the downregulation of DHX58 expression and alleviate I/R injury, and this effect is dependent on DHX58 (Jia et al., 2024). This study first revealed that DHX58 inhibits hepatocyte ferroptosis through m6A-dependent translational regulation, thereby mitigating I/R injury, and also expands the known non-immune functions of DHX58. This finding provides a new perspective for understanding the molecular mechanisms of liver I/R injury and lays a theoretical foundation for developing ferroptosis-targeted liver protection strategies.

During HIRI progression, the expression of the m6A demethylase FTO is significantly decreased. However, overexpression of FTO can markedly reduce the extent of liver tissue necrosis, lower the levels of alanine aminotransferase (ALT) and aspartate aminotransferase (AST), enhance the liver’s antioxidant capacity, and effectively inhibit mitochondrial fragmentation. Mechanistically, FTO achieves this by removing the methylation modification on dynamin-related protein 1 (*Drp1*) mRNA, reducing its stability and protein expression, thereby blocking Drp1-mediated excessive mitochondrial fission (Du et al., 2021).

Following liver I/R, both overall m6A levels and the expression of methyltransferase-like 3 (METTL3) are significantly elevated, peaking particularly at 6 h of reperfusion. Hepatocyte-specific METTL3 deficiency aggravates liver injury and increases ERK and JNK phosphorylation, whereas AAV-mediated METTL3 overexpression reduces serum ALT/AST levels, tissue necrosis, TUNEL-positive cells, and pro-inflammatory factor expression, indicating a protective role for METTL3 in this model (Gao et al., 2024).

Research by Guo et al. (2025) showed that NIPSNAP1 expression is significantly downregulated during I/R. Knocking down NIPSNAP1 expression impairs mitophagy, exacerbates mitochondrial fission, and leads to more severe liver injury. The m6A reader protein insulin-like growth factor 2 mRNA-binding protein 2 (IGF2BP2) can directly bind to *NIPSNAP1* mRNA and enhance its stability. Overexpression of IGF2BP2 can improve mitochondrial homeostasis and alleviate injury by upregulating NIPSNAP1 expression, but this effect is blocked by silencing NIPSNAP1 (Guo et al., 2025). ALKBH5 has also been reported to protect against hepatic I/R injury. In mouse hepatic I/R and hepatocyte H/R models, ALKBH5 overexpression increased YTHDF1 mRNA stability and thereby promoted YAP translation, supporting hepatocyte proliferation and reducing injury (Wang et al., 2024).

A study integrated two liver transplantation gene expression datasets (GSE12720 and GSE151648) from the GEO database. Using three machine learning algorithms—least absolute shrinkage and selection operator (LASSO), random forest, and support vector machine recursive feature elimination (SVM-RFE)—for cross-screening, the study ultimately identified YTHDC1, METTL3, WTAP, and NUDT3 as core genes. These genes not only demonstrated good diagnostic efficacy but also showed significant correlations with the infiltration levels of various immune cells in liver tissue, such as activated CD4+ T cells, neutrophils, and mast cells (Meng et al., 2024).

Analysis of the gene expression profile from the GSE23649 dataset revealed that WTAP, CBLL1, RBM15, and YTHDC1 were upregulated after reperfusion, while LRPPRC, FTO, METTL3, and ALKBH5 were downregulated. A random forest (RF) model successfully screened eight key regulatory factors and constructed a well-calibrated nomogram prediction model. Further analysis indicated that these factors were significantly correlated with the infiltration of various immune cells, including activated T cells, dendritic cells, and neutrophils (Zhang et al., 2023).

Joint analysis of methylated RNA immunoprecipitation sequencing (MeRIP-seq) and RNA sequencing (RNA-seq) on mouse hepatic I/R models and sham-operated groups depicted a transcriptome-wide m6A methylation map. The results showed that the number of m6A peaks in the I/R group (21,557) was significantly higher than in the control group (16,917), and the differential peaks were mainly concentrated in the coding sequence (CDS) and 3′ untranslated region (3′UTR). Through integrated analysis, three overlapping genes—Fnip2, Phldb2, and Pcf11—that were significantly upregulated in both m6A methylation level and mRNA expression were successfully identified (Hua et al., 2023).

In summary, these studies not only reveal specific regulatory axes such as FTO-Drp1, METTL3-MAPK, IGF2BP2-NIPSNAP1, and ALKBH5-YTHDF1-YAP but also utilize bioinformatics methods to construct an association network between m6A modification and the immune microenvironment. Furthermore, they provide a comprehensive profiling of m6A modification patterns in hepatic ischemia-reperfusion injury.

#### Regulation and mechanisms of m6A modification in renal ischemia-reperfusion injury

In the field of renal ischemia-reperfusion injury (IRI), research both domestically and internationally has yielded a series of significant findings. Studies have revealed multiple key regulatory axes and their roles in processes such as pyroptosis, apoptosis, autophagy, and ferroptosis.

At the cellular level, hypoxia/reoxygenation (H/R) induces severe damage in HK-2 cells, accompanied by an increase in overall m6A methylation levels and decreased FTO expression. Within this process, FTO mediates m6A demethylation to reduce the m6A modification on *Ambra1* mRNA, thereby restoring Ambra1 expression and activating ULK1-mediated autophagy, exerting a protective effect against IRI (Chen et al., 2024). WTAP expression increases during renal I/R injury, and it may promote cell apoptosis by enhancing the m6A modification of *FOXO1* and consequently its expression (Zhao et al., 2023).

Under IRI conditions, METTL3 expression is significantly upregulated, overall m6A levels are elevated, and renal tissue damage is more severe. Differential modification genes such as *Foxd1*, *Tfap2a*, and *Cyp1b1* were identified *via* MeRIP-seq, among which the m6A modification level of *Foxd1* showed a negative correlation with its mRNA expression and demonstrated the best consistency in methylation and expression assays. METTL3 knockdown reduced H/R-induced apoptosis, while Foxd1 showed concordant methylation and expression changes (Meng et al., 2020).

In renal tissue from acute kidney injury (AKI) patients and in mouse IRI models, both m6A levels and METTL14 expression are significantly elevated and correlated with renal function indicators. METTL14 enhances the m6A modification of *YAP1* mRNA, promoting its degradation and suppressing YAP1 expression. This study is the first to identify *YAP1* as a direct target of METTL14 (Xu et al., 2020b).

FTO can reduce the m6A modification on *Ambra1* mRNA, restoring its expression, thereby activating ULK1-mediated autophagy. SP1, acting as an upstream transcription factor, directly binds to the FTO promoter and promotes its transcription (Chen et al., 2024). In renal I/R injury, PPARγ expression is significantly downregulated. Research found that METTL14-mediated m6A methylation of *PPARγ* mRNA, dependent on the reader protein IGF2BP2, enhances *PPARγ* mRNA stability and upregulates its expression (Liu et al., 2024c).

After METTL3 enhances the m6A modification on *TAB3* mRNA, it recruits the reader protein IGF2BP2 to increase its stability, activating the NF-κB pathway, exacerbating inflammation and cell death. Inhibiting METTL3 can alleviate renal injury (Wang et al., 2022b). ALKBH5 deficiency can mitigate renal function impairment and histopathological changes. ALKBH5 removes m6A modification from its direct target *Ccl28* mRNA, reducing IGF2BP2-mediated stability and inhibiting CCL28 expression. Upregulation of CCL28 promotes regulatory T cell (Treg) recruitment, suppresses neutrophil and macrophage infiltration, and fosters a protective immune microenvironment (Chen et al., 2023).

Yao et al. (2025) integrating evidence from numerous animal models, cellular experiments, and clinical samples, constructed a comprehensive regulatory network encompassing “writers,” “erasers,” and “readers.” Their study found that m6A dysregulation is widespread but manifests diversely. For instance, METTL3 plays a pro-injury role in most kidney diseases; the function of FTO exhibits strong cell-type dependency, exacerbating lipotoxicity in podocytes but exerting anti-inflammatory effects in macrophages (Yao et al., 2025).

PGC-1α-driven mitochondrial biogenesis is an established protective mechanism in renal IRI more broadly (Li et al., 2025). The only direct evidence linking FTO to this axis, however, comes from clear cell renal cell carcinoma (ccRCC). In ccRCC tissues, FTO is significantly downregulated, and its low expression is closely associated with tumor progression and poor prognosis. Overexpression of FTO can inhibit tumor growth and induce oxidative stress, an effect dependent on its demethylase activity. FTO reduces the m6A modification level of *PGC-1α* mRNA, enhancing its stability and expression, thereby activating mitochondrial biogenesis (Zhuang et al., 2019). This ccRCC-derived mechanism is presented here as a testable hypothesis for renal IRI rather than as established IRI evidence.

#### Cross-organ comparative synthesis

The organ-specific findings summarized above (“Regulatory Mechanisms of m6A in Myocardial Ischemia-Reperfusion Injury”-“Regulation and Mechanisms of m6A Modification in Renal Ischemia-Reperfusion Injury”) reveal recurring patterns when compared directly. Cross-organ comparison indicates that the net effect of an m6A regulator depends on the target transcript and organ context, that reader context may contribute to divergent RNA outcomes, and that several m6A-regulated pathways converge on mitochondrial quality control. Tables 1 and 2 summarize the corresponding organ-specific axes and target-level evidence.

**Table 1:** Organ-specific m6A regulatory axes in ischemia-reperfusion injury. “Proximal target” denotes an RNA supported by target-level m6A or regulator-binding assays in the cited study, whereas “downstream” denotes subsequent effectors or phenotypes. Evidence type describes the support reported in the cited study: mechanistic, mechanistic with an unresolved target, profiling/association, bioinformatic, intervention/delivery, or contextual review. Effects refer to increased expression or activity of the listed regulator in the indicated model.

Organ	Regulator/model	Change	Proximal m6A-associated RNA	Reader/downstream	Outcome	Net effect	Evidence; ref.
Heart	WTAP/YTHDF2; mouse I/R, cardiomyocyte H/R	WTAP activity	Snhg1	miR-361-5p/OPA1	Mitochondrial fusion reduced	Detrimental	Mechanistic; Liu et al. (2024a)
Heart	METTL14; myocardial I/R (knockdown study)	↑ after I/R; knockdown ↓ injury	Not identified	Akt/mTOR-associated	Knockdown reduces apoptosis, necrosis, ROS, and inflammatory indices	METTL14 activity detrimental in model	Mechanistic (target unresolved); Wu et al. (2024)
Heart	YTHDF2; rat I/R	NR	BNIP3	Excessive mitophagy restrained	Smaller infarct, improved function	Protective	Mechanistic; Cai et al. (2023)
Heart	METTL3; rat I/R, OGD/R	↑	pri-miR-143-3p	DGCR8/miR-143-3p/PRKCE; pyroptosis markers downstream	Pyroptosis	Detrimental	Mechanistic; Wang et al. (2023b)
Heart	FTO; mouse I/R tissue; cardiomyocyte H/R	↓	YAP1 mRNA	YAP1 expression	Apoptosis and inflammation reduced in H/R cells	Protective	Mechanistic (H/R cells); Ke et al. (2022)
Heart	WTAP/YTHDF1; rat I/R, H9C2 H/R	WTAP ↑	FOXO3a mRNA	YTHDF1-dependent stability	Apoptosis and inflammation	Detrimental	Mechanistic; Wang et al. (2023a)
Heart	METTL3/IGF2BP2; mouse I/R, HL-1 H/R	NR	Klf6 mRNA	KLF6→Acsl4	Ferroptosis	Detrimental	Mechanistic; Qiu, Yan & Liu (2023)
Heart	METTL14; HL-1 H/R	↑	pri-miR-146a-5p	DGCR8/miR-146a-5p→APPL1	Ferroptosis	Detrimental	Mechanistic; Zhao & Li (2024)
Heart	ALKBH5; rat MIRI, H9C2 H/R	NR	Ythdf1 mRNA	YTHDF1→Fth1 translation	Ferroptosis reduced	Protective	Mechanistic; Yin & Liu (2025)
Heart	ALKBH5 inhibition; AMI/I/R-associated model	Inhibited	Not identified	IOX1 nanocage	Infarct reduced; function improved	Protective intervention	Intervention/delivery study; Cheng et al. (2022)
Brain	FTO; mouse/rat CIRI profiling	↓	Not identified	Global m6A ↑	Descriptive injury association	Not assignable	Profiling/association; Chokkalla et al. (2019; Shao et al. (2022)
Brain	FTO; rat MCAO/R plus acupuncture	↑ after treatment	ACSL4 mRNA	ACSL4/ferroptosis	Infarct and ferroptosis reduced	Protective	Intervention/delivery study; Liu et al. (2026)
Brain	METTL3; rat CIRI plus eye acupuncture	↑ after treatment	Not identified	HIF-1α/VEGF-A-associated	Angiogenesis; neurological measures improved	Protective association	Intervention/delivery study; Pu, Wang & Chu (2025)
Brain	ALKBH5/FTO; CIRI models	ALKBH5 ↑; FTO ↓	Not directly established	Bcl2 stability	Apoptosis reduced	Protective	Regulator perturbation; target demethylation inferred; Xu et al. (2020a)
Brain	METTL3/YTHDC1; rat MCAO, OGD/R	NR	CRNDE lncRNA	YTHDC1/ATG10	Autophagy; apoptosis/inflammation reduced	Protective	Mechanistic; Yu et al. (2024)
Brain	METTL14; mouse MCAO/R, neurons OGD/R	NR	FDX1 mRNA	FDX1/cuproptosis	Neuronal cuproptosis	Detrimental	Mechanistic; Ding et al. (2025)
Brain	YTHDF1; mouse MCAO/R, HT22 OGD/R	↑	p53 mRNA	p53 translation; SLC7A11/GPX4 downstream	Ferroptosis	Detrimental	Mechanistic; Chang et al. (2025)
Brain	METTL3; mouse MCAO/R, N2a OGD/R	NR	AU020206 lncRNA	AU020206-YTHDC2; SLC7A11/GPX4 downstream	Apoptosis and ferroptosis reduced by METTL3	Protective	Mechanistic; Zhang et al. (2025)
Brain	METTL3; mouse MCAO/R, astrocyte OGD/R	NR	ABHD11-AS1 lncRNA	miR-1301-3p/HIF1AN/HIF-1α-Nox4	Neural injury	Detrimental	Mechanistic; Huang et al. (2024)
Brain	METTL3; rat MCAO/R, neuronal OGD/R	NR	pri-miR-335	miR-335→eRF1; stress granules	Apoptosis reduced	Protective, early phase	Mechanistic; Si et al. (2020)
Brain	METTL3/catalpol; cerebral ischemia, OGD/R	Treatment-associated	Beclin-1 mRNA	NRF1/KAT2A/METTL3	Excessive autophagy reduced	Protective association	Mechanistic; Liu et al. (2024d)
Brain	m6A-associated; mouse MCAO/R, endothelial OGD/R	m6A ↑	MMP3 mRNA	CBLL1 association	Blood-brain barrier disruption	Detrimental	Profiling/association; Liang et al. (2023)
Liver	DHX58/YTHDC2; partial I/R, hepatocyte H/R	DHX58 ↓	Gpx4 mRNA	YTHDC2-dependent translation	Ferroptosis reduced when restored	Protective	Mechanistic; Jia et al. (2024)
Liver	FTO; mouse I/R, hepatocyte H/R	↓	Drp1 mRNA	Drp1 stability/expression ↓	Mitochondrial fragmentation reduced	Protective	Mechanistic; Du et al. (2021)
Liver	METTL3; hepatocyte-specific mouse I/R, L02 H/R	↑	Not identified	ERK/JNK activation limited	Necrosis, enzymes, inflammation reduced	Protective	Mechanistic (target unresolved); Gao et al. (2024)
Liver	IGF2BP2; mouse I/R, HepG2 H/R	NR	NIPSNAP1 mRNA	IGF2BP2-dependent stability	Mitophagy and mitochondrial dynamics	Protective	Mechanistic; Guo et al. (2025)
Liver	ALKBH5; warm I/R, L02 H/R	NR	YTHDF1 mRNA	YTHDF1→YAP translation	Liver injury reduced	Protective	Mechanistic; Wang et al. (2024)
Liver	Mixed methylation-gene panels; transplant datasets	Dataset-specific	Not established	Inferred immune associations	Candidate signatures	Not assignable	Bioinformatic; Meng et al. (2024; Zhang et al. (2023)
Liver	MeRIP/RNA-seq mapping; mouse I/R	Peak count ↑	Fnip2/Phldb2/Pcf11 candidates	No named regulator	Candidate target map	Not assignable	Profiling/association; Hua et al. (2023)
Kidney	FTO; rat I/R, HK-2 H/R	↓	Ambra1 mRNA	Ambra1/ULK1 autophagy	Oxidative stress and apoptosis reduced	Protective	Mechanistic; Chen et al. (2024)
Kidney	WTAP; mouse I/R, HK-2 H/R	↑	FOXO1 mRNA	FOXO1-associated apoptosis	Renal injury	Detrimental	Mechanistic; Zhao et al. (2023)
Kidney	METTL3; rat I/R, tubular H/R	↑	Foxd1 mRNA	Foxd1 suppression	Renal injury	Detrimental	Regulator perturbation; target association; Meng et al. (2020)
Kidney	METTL14; renal I/R/HK-2	↑	YAP1 mRNA	YAP1 suppression	Renal injury	Detrimental	Mechanistic; Xu et al. (2020b)
Kidney	METTL14/IGF2BP2; mouse I/R	NR	PPARγ mRNA	IGF2BP2-dependent stability	Tubular ferroptosis reduced	Protective	Mechanistic; Liu et al. (2024c)
Kidney	METTL3/IGF2BP2; multi-model AKI including I/R	NR	TAB3 mRNA	TAB3/NF-κB inflammation	Renal inflammation and injury	Detrimental	Mechanistic; Wang et al. (2022b)
Kidney	ALKBH5; male mouse I/R	NR	Ccl28 mRNA	IGF2BP2 stability; Treg recruitment ↓	Inflammation	Detrimental	Mechanistic; Chen et al. (2023)

**Note:**

“Proximal target” denotes an RNA supported by target-level m6A or regulator-binding assays in the cited study, whereas “downstream” denotes subsequent effectors or phenotypes. Evidence type describes the support reported in the cited study: mechanistic, mechanistic with an unresolved target, profiling/association, bioinformatic, intervention/delivery, or contextual review. Effects refer to increased expression or activity of the listed regulator in the indicated model.

**Table 2:** Target-resolved m6A-associated RNAs in ischemia–reperfusion injury. “Target-level evidence” refers to the experimental support for the indicated m6A-associated RNA in the cited study. Downstream effectors are reported in a separate column.

Target RNA	RNA class	Organ/model	Upstream effector	Target-level evidence	Downstream consequence	Ref.
Snhg1	lncRNA	Heart I/R; cardiomyocyte H/R	WTAP/YTHDF2	m6A-associated destabilization	miR-361-5p/OPA1; mitochondrial fusion	Liu et al. (2024a)
pri-miR-143-3p	pri-miRNA	Heart I/R; OGD/R	METTL3/DGCR8	m6A-facilitated processing	miR-143-3p→PRKCE; pyroptosis	Wang et al. (2023b)
FOXO3a mRNA	mRNA	Heart I/R; H/R	WTAP/YTHDF1	m6A-associated stability	Apoptosis and inflammation increased	Wang et al. (2023a)
BNIP3 mRNA	mRNA	Heart I/R	YTHDF2	Reader-associated downregulation	Excessive mitophagy restrained	Cai et al. (2023)
Klf6 mRNA	mRNA	Heart I/R; H/R	METTL3/IGF2BP2	m6A-associated stability	KLF6→Acsl4; ferroptosis	Qiu, Yan & Liu (2023)
pri-miR-146a-5p	pri-miRNA	Cardiomyocyte H/R	METTL14/DGCR8	m6A-facilitated processing	miR-146a-5p→APPL1; ferroptosis	Zhao & Li (2024)
Ythdf1 mRNA	mRNA	Rat MIRI; H9C2 H/R	ALKBH5	Demethylation/expression	YTHDF1→Fth1 translation; ferroptosis reduced	Yin & Liu (2025)
ACSL4 mRNA	mRNA	Rat cerebral I/R with acupuncture	FTO	m6A-associated regulation	Ferroptosis reduced	Liu et al. (2026)
Bcl2 mRNA	mRNA	Cerebral I/R models	ALKBH5/FTO	Stability assays; demethylation inferred	Apoptosis reduced	Xu et al. (2020a)
CRNDE	lncRNA	Rat MCAO; SH-SY5Y OGD/R	METTL3/YTHDC1	m6A-associated regulation	ATG10/autophagy; injury reduced	Yu et al. (2024)
FDX1 mRNA	mRNA	Mouse MCAO/R; neuronal OGD/R	METTL14	m6A-associated stability	Cuproptosis increased	Ding et al. (2025)
p53 mRNA	mRNA	Mouse MCAO/R; HT22 OGD/R	YTHDF1	m6A-dependent binding/translation	SLC7A11/GPX4 downstream; ferroptosis	Chang et al. (2025)
AU020206	lncRNA	Mouse MCAO/R; N2a OGD/R	METTL3	m6A-associated degradation	AU020206-YTHDC2; SLC7A11/GPX4 downstream; apoptosis and ferroptosis reduced	Zhang et al. (2025)
ABHD11-AS1	lncRNA	Mouse MCAO/R; astrocyte OGD/R	METTL3	m6A-associated stability	miR-1301-3p/HIF1AN/HIF-1α-Nox4; neural injury increased	Huang et al. (2024)
pri-miR-335	pri-miRNA	Rat MCAO/R; neuronal OGD/R	METTL3	m6A-facilitated processing	miR-335→eRF1; stress granules; apoptosis reduced	Si et al. (2020)
Beclin-1 mRNA	mRNA	Cerebral ischemia; OGD/R	METTL3/catalpol pathway	m6A-associated regulation	Excessive autophagy reduced	Liu et al. (2024d)
MMP3 mRNA	mRNA	Mouse MCAO/R; endothelial OGD/R	m6A-associated; writer unresolved	MeRIP-level association	Blood-brain barrier disruption	Liang et al. (2023)
Gpx4 mRNA	mRNA	Liver I/R; hepatocyte H/R	DHX58/YTHDC2	Binding and reader recruitment	GPX4 translation; ferroptosis reduced	Jia et al. (2024)
Drp1 mRNA	mRNA	Liver I/R; H/R	FTO	Demethylation/stability	Mitochondrial fission reduced	Du et al. (2021)
NIPSNAP1 mRNA	mRNA	Liver I/R; HepG2 H/R	IGF2BP2	m6A-dependent binding/stability	Mitophagy and mitochondrial dynamics preserved	Guo et al. (2025)
YTHDF1 mRNA	mRNA	Liver I/R; L02 H/R	ALKBH5	Demethylation/stability	YTHDF1→YAP translation; liver injury reduced	Wang et al. (2024)
Ambra1 mRNA	mRNA	Renal I/R; HK-2 H/R	FTO	Demethylation/expression	ULK1-associated autophagy; oxidative stress and apoptosis reduced	Chen et al. (2024)
FOXO1 mRNA	mRNA	Renal I/R; HK-2 H/R	WTAP	m6A-associated regulation	Apoptosis increased	Zhao et al. (2023)
Foxd1 mRNA	mRNA	Renal I/R; tubular H/R	METTL3	MeRIP-supported methylation	Foxd1 suppression; injury mediation unconfirmed	Meng et al. (2020)
YAP1 mRNA	mRNA	Renal I/R; HK-2	METTL14	m6A-associated suppression	Renal injury	Xu et al. (2020b)
PPARγ mRNA	mRNA	Mouse renal I/R	METTL14/IGF2BP2	m6A-associated stability	Tubular ferroptosis reduced	Liu et al. (2024c)
TAB3 mRNA	mRNA	Multi-model AKI including I/R	METTL3/IGF2BP2	m6A-associated stability	NF-κB inflammation	Wang et al. (2022b)
Ccl28 mRNA	mRNA	Male mouse renal I/R	ALKBH5/IGF2BP2	Demethylation/stability	Treg recruitment reduced; inflammation increased	Chen et al. (2023)

**Note:**

“Target-level evidence” refers to the experimental support for the indicated m6A-associated RNA in the cited study. Downstream effectors are reported in a separate column.

METTL3 illustrates this paradox most clearly. In cardiac IRI, METTL3 upregulation promotes pyroptosis *via* the NLRP3–caspase-1–GSDMD-N axis, and in renal IRI it promotes injury through IGF2BP2-dependent TAB3/NF-κB activation. Yet within cerebral IRI, the reported role of METTL3 is itself inconsistent: METTL3 overexpression has been reported to be protective, suppressing lncRNA CRNDE and reducing apoptosis, and to promote stress-granule assembly *via* pri-miR-335 with an anti-apoptotic effect, while a separate study reports that METTL3 deficiency—implying a detrimental role for METTL3—is neuroprotective *via* the ABHD11-AS1/miR-1301-3p/HIF1AN axis. This indicates that METTL3’s functional output is genuinely target-transcript-dependent even within a single organ, more plausibly reflecting differences in basal m6A landscape, reader-protein expression, and target-transcript spectra across cell types than a fixed property of the enzyme itself.

A second recurring pattern involves convergent regulation of a single downstream node by different m6A enzymes in different organs. YAP1/YAP is one example: FTO-mediated YAP1 mRNA stabilization was linked to reduced apoptosis in H/R-treated cardiomyocytes (Ke et al., 2022). METTL14 destabilizes YAP1 mRNA in the kidney, where this loss of YAP1 is associated with worse injury; and ALKBH5 regulates YAP *via* YTHDF1 in the liver, where it is protective. The opposing effects of YTHDF2 and IGF2BP proteins suggest that reader context may influence the fate of m6A-marked transcripts; however, competition at the same site has not been demonstrated in IRI models. YTHDF2 recruits the CCR4–NOT complex to accelerate decay of its targets, whereas IGF2BP proteins recognize m6A to stabilize their targets—a functionally opposite outcome from the same modification. Within the studies reviewed here, IGF2BP2 stabilizes NIPSNAP1 in the liver to preserve mitophagy and PPARγ in the kidney to limit injury, while YTHDF1 promotes m6A-dependent p53 translation in the brain, leading to downstream suppression of the SLC7A11/GPX4 axis and increased ferroptosis. The relative expression of decay-promoting (YTHDF2) *vs*. stability-promoting (IGF2BP1–3) readers in a given cell type may be a decisive, testable determinant of whether a given m6A event is ultimately protective or detrimental. Testing this hypothesis will require matched reader-occupancy and perturbation experiments in the same cell type and reperfusion window.

A third convergent theme is mitochondrial quality control, which m6A-associated regulation affects at several levels: mitochondrial fusion (WTAP–Snhg1–miR-361-5p–OPA1 in the heart), excessive fission (FTO–Drp1 in the liver), and mitophagy control (YTHDF2 restrains BNIP3-associated excessive mitophagy in the heart, whereas IGF2BP2 stabilizes NIPSNAP1 to preserve mitophagy and mitochondrial dynamics in the liver). The ccRCC FTO–PGC-1α finding remains a non-IRI hypothesis for mitochondrial biogenesis.

Finally, cross-organ comparison is constrained by a temporal limitation common to nearly all of the studies above: m6A and its regulators were measured at a single, study-specific time point after reperfusion, rather than as a genuine time course. Direct quantitative comparison across organs should therefore be treated as qualitative rather than strictly time-matched. The available evidence is therefore weighted toward acute reperfusion, while recovery and long-term remodeling remain understudied.

## Discussion

Evidence across distinct organ systems indicates that m6A-associated regulation contributes to the pathological trajectory of ischemia-reperfusion damage. Investigations spanning cerebral, hepatic, renal, and cardiac tissues report model-dependent m6A changes during IRI, with increased methylation observed in several models. These epitranscriptomic alterations have been associated with increased expression or activity of methyltransferases, including METTL3, METTL14, and WTAP, or reduced activity of demethylases like FTO and ALKBH5. By precisely regulating the mRNA stability, translation efficiency, or splicing of specific target genes, m6A modification influences cell fate.

In cardiac IRI, m6A participates in regulating signaling axes such as Akt/mTOR (Wu et al., 2024), FOXO3a (Wang et al., 2023a), YAP1 (Ke et al., 2022), and Klf6 (Qiu, Yan & Liu, 2023), and is involved in various forms of programmed cell death in cardiomyocytes, including apoptosis (Liu et al., 2024b; Wang et al., 2023a), pyroptosis (Wang et al., 2023b), ferroptosis (Zhao & Li, 2024), and mitophagy (Cai et al., 2023). In cerebral IRI, m6A not only regulates neuroinflammation, autophagy, and blood-brain barrier integrity (Liu et al., 2024d; Liang et al., 2023) but has also been found to participate in novel cell death modalities such as ferroptosis (Liu et al., 2026; Chang et al., 2025) and cuproptosis (Ding et al., 2025). In hepatic IRI, m6A regulates mitochondrial dynamics and ferroptosis through axes like FTO-Drp1 (Du et al., 2021), IGF2BP2-NIPSNAP1 (Guo et al., 2025), and DHX58-YTHDC2-GPX4 (Jia et al., 2024). ALKBH5-YTHDF1-YAP signaling has additionally been linked to hepatocyte proliferation and reduced liver injury (Wang et al., 2024). In renal IRI, m6A is extensively involved in inflammation, apoptosis, pyroptosis, autophagy, and ferroptosis by regulating pathways such as TAB3/NF-κB (Wang et al., 2022b), PPARγ (Liu et al., 2024c), FOXO1 (Zhao et al., 2023), YAP1 (Xu et al., 2020b), and Ambra1 (Chen et al., 2024). Together, these findings identify m6A-associated regulation as a recurrent component of IRI pathophysiology across organs.

Despite these mechanistic insights, organ-specific functional disparities prevent the establishment of a universal m6A regulatory model. Specifically, while METTL3 amplifies pathological damage in both cardiac (Wang et al., 2023b) and renal (Wang et al., 2022b) tissues, this same methyltransferase paradoxically confers neuroprotection in specific cerebral ischemia paradigms (Yu et al., 2024; Si et al., 2020). Such contradictory observations likely arise from heterogeneous cell populations and varying experimental models, underscoring the necessity for comprehensive cross-organ evaluations. Furthermore, most studies remain at the stage of animal models or cell experiments, with very limited data translatable to the clinic. Although some studies have attempted to construct nomogram prediction models (Wang et al., 2022c; Zhang et al., 2023) or explore intervention methods such as acupuncture (Liu et al., 2026; Pu, Wang & Chu, 2025) and nano-delivery (Cheng et al., 2022), we did not identify an IRI clinical trial of a therapeutic strategy directly targeting m6A among the literature included in this review. The METTL3 inhibitor STC-15 has entered a first-in-human phase 1 trial in patients with advanced malignancies (Moser et al., 2024). Because METTL3 appears protective in some cerebral contexts but detrimental in cardiac and renal IRI, a systemically administered METTL3 inhibitor could plausibly help one organ while harming another; organ-selective delivery is therefore likely to be a prerequisite for translating m6A-targeted therapeutics to IRI. Clinical translation is further constrained by systemic exposure, off-target effects, uncertain dose windows, and the lack of human I/R validation. Additionally, the precise mechanisms of action of m6A reader proteins are not fully understood. While the functions of the YTHDF and IGF2BP families have been preliminarily investigated, the roles of other readers (*e.g*., YTHDC1/2) remain unclear. Moreover, the same reader protein may exhibit opposing functions in different tissues, indicating that its regulation is highly context-dependent. The absence of archived database-specific search histories and record counts limits the reproducibility of the literature search. A further limitation is methodological: a substantial proportion of mechanistic evidence reviewed here relies on antibody-based MeRIP-seq, which lacks single-nucleotide resolution and has limited sensitivity for low-abundance transcripts. Emerging approaches include nanopore direct RNA sequencing and antibody-independent chemical-labelling methods (Moshitch-Moshkovitz et al., 2024). No application of these approaches to an IRI model was identified among the studies included in this review. Concurrently, research on the interactions between m6A modification and other epigenetic factors is scarce, limiting our understanding of the overall regulatory network. Finally, the mechanisms by which m6A responds to metabolic reprogramming or changes in the immune microenvironment (*e.g*., Treg infiltration) (Chen et al., 2023) require further investigation. In summary, although substantial achievements have been made in m6A research related to IRI, challenges remain, including insufficient mechanistic depth, weak clinical connections, and an incomplete understanding of regulatory networks.

## Future directions

Building on the gaps identified above, we highlight four priorities for the field. First, spatial and single-cell epitranscriptomic profiling is needed to resolve whether the organ- and cell-type-specific outcomes described here reflect genuinely distinct m6A landscapes rather than bulk-tissue averaging artifacts (Feng & Koziol, 2026; Tegowski et al., 2024). Second, multi-timepoint study designs spanning the ischemic, early-reperfusion, and late remodeling phases within a single model are needed to establish true time courses of m6A dynamics. Third, multi-omics integration that combines m6A profiling with proteomic and metabolomic data would help establish which m6A-associated transcriptional changes translate into functional consequences. Fourth, translating m6A-targeted compounds to IRI will require organ-selective delivery strategies capable of achieving the organ-specific, and in some cases organ-opposite, modulation that this review’s synthesis indicates will be required. These approaches will require standardized I/R models, matched reader-occupancy and perturbation experiments, and clinically annotated human tissues before translational claims can be made.

## Conclusion

Across hepatic, renal, cerebral, and cardiac systems, m6A RNA methylation contributes to IRI pathogenesis, but does so through organ-specific, and in several cases directly conflicting, regulatory logic rather than a single universal mechanism: the same regulator (*e.g*., METTL3, ALKBH5) can be protective in one organ and detrimental in another. This organ specificity may reflect differences in reader-protein expression and target-transcript spectra rather than an intrinsic property of any single writer, eraser, or reader. Mechanistically, the evidence converges on mitochondrial quality control, cell-death execution, and inflammatory signaling as the principal downstream axes through which m6A shapes IRI outcomes. Most of this evidence, however, derives from preclinical models with limited temporal and cell-type resolution, and many studies rely on antibody-based methylation profiling; translating these findings into clinical applications will require time-resolved, single-cell-resolution mechanistic studies, base-resolution detection methods, human-relevant validation, and organ-selective delivery platforms.

## Additional Information and Declarations

### Data Availability

The following information was supplied regarding data availability:

This is a Literature Review.
